# Intake of Lutein and Zeaxanthin as a Possible Factor Influencing Gastrointestinal Symptoms in Caucasian Individuals with Ulcerative Colitis in Remission Phase

**DOI:** 10.3390/jcm8010077

**Published:** 2019-01-11

**Authors:** Dominika Głąbska, Dominika Guzek, Paulina Zakrzewska, Gustaw Lech

**Affiliations:** 1 Department of Dietetics, Faculty of Human Nutrition and Consumer Sciences, Warsaw University of Life Sciences (SGGW-WULS), 159c Nowoursynowska Street, 02-776 Warsaw, Poland; paulina_zakrzewska@sggw.pl; 2 Department of Organization and Consumption Economics, Faculty of Human Nutrition and Consumer Sciences, Warsaw University of Life Sciences (SGGW-WULS), 159c Nowoursynowska Street, 02-776 Warsaw, Poland; dominika_guzek@sggw.pl; 3 Department of General, Gastroenterological and Oncological Surgery, Medical University of Warsaw, 1a Banacha Street, 02-097 Warsaw, Poland; gustaw.lech@wum.edu.pl

**Keywords:** colitis ulcerosa, gastrointestinal symptoms, constipation, lutein, zeaxanthin

## Abstract

The vitamin A deficiencies are commonly observed in the case of ulcerative colitis individuals. The decreased antioxidant defence may influence the intestine, inducing higher susceptibility to oxidative damage of tissues and altering the symptoms and course of disease. Intestinal symptoms, ranging from diarrhea to constipation, occur more commonly in remission ulcerative colitis individuals than in general population. The aim of the study was to analyze the association between retinoid intake and gastrointestinal symptoms in Caucasian individuals in the remission phase of ulcerative colitis. Reitnoid (total vitamin A, retinol, β-carotene, α-carotene, β-cryptoxanthin, lycopene, as well as lutein and zeaxanthin) intakes were analyzed on the basis of three-day dietary records. Gastrointestinal symptoms (daily number of bowel movements, and the presence of painful tenesmus, flatulence, and constipation) were self-reported. The study was conducted in a group of 56 ulcerative colitis remission individuals, stratified by the gastrointestinal symptoms. One in every seven individuals reported recurring constipation. Higher intake of lutein and zeaxanthin (median 1386.2 µg, 289.0–13221.3 µg vs. median 639.0 µg, 432.7–1309.0 µg) may lower the incidence of constipation (*p* = 0.013). The intake of retinoids other than lutein and zeaxanthin was not associated with the incidence of constipation or other gastrointestinal symptoms.

## 1. Introduction

In the European Prospective Investigation into Cancer and Nutrition, which was conducted in ten European Union countries, it was confirmed that retinoids were potentially beneficial for colon. Plasma retinol concentration and β-carotene intake have both been reported to be inversely correlated with the risk of colon cancer [1]. This effect is attributed to the antioxidative properties of different forms of vitamin A [2]. A meta-analysis of randomized controlled trials revealed that these antioxidative properties of vitamin A promote reduction of oxidative stress [3]. Therefore, the intake of vitamin A may be of a great value, especially for individuals with inflammatory bowel diseases, as oxidative stress contributing to carcinogenesis [4] is one of the factors commonly observed in patients with ulcerative colitis and Crohn’s disease [5].

An increase in the level of oxidative stress has been recognized to occur independent of the activity of ulcerative colitis [6]. This increase in oxidative stress may be associated with an increased turnover of nutrients [7]. Moreover, a lower absorption of retinoids, which are fat-soluble compounds [8], may be observed in patients with inflammatory bowel diseases due to impaired fat digestion and lower fat absorption [9,10].

As a result of poor absorption, vitamin A deficiencies are commonly observed in individuals with inflammatory bowel diseases [11]. In addition, vitamin A intake is generally lower than recommended in individuals with ulcerative colitis. A reduced intake of vitamin A was observed in 40% of patients with ulcerative colitis [12] and 26% of patients with inflammatory bowel diseases in previous studies [13]. As a result of reduced intake, decreased antioxidant defense may affect the intestine, inducing higher susceptibility to oxidative damage of tissues, as well as hindering the recovery of the mucosa and improvement of epithelial cell layer integrity, both of which are observed in individuals with inflammatory bowel diseases [14]. Based on these findings, retinoids are considered to influence the symptoms and course of the ulcerative colitis [15], but taking it into account, especially valuable may be retinoids, which are characterized by the strong antioxidant activity, such as lutein and zeaxanthin [16].

The symptoms of inflammatory bowel diseases, including altered intestinal motility and functioning, may be observed even during remission, due to the course of the disease and psychological factors [17]. Intestinal symptoms, ranging from diarrhea with fecal incontinence to constipation, are observed to occur more commonly in groups of individuals in the remission phase of inflammatory bowel diseases than in the general population [18]. Moreover, these intestinal symptoms may modulate other symptoms of inflammatory bowel diseases [19] and impair the general quality of life of the affected patients [20]. Hence, the effective treatment of intestinal symptoms would be of a great significance to improve the functioning and well-being of patients with inflammatory bowel diseases.

The aim of the study was to analyze the association between retinoid intake (total vitamin A, retinol, β-carotene, α-carotene, β-cryptoxanthin, lycopene, as well as lutein and zeaxanthin) and self-reported gastrointestinal symptoms (daily number of bowel movements, and the presence of painful tenesmus, flatulence, and constipation) in Caucasian individuals in the remission phase of ulcerative colitis.

## 2. Experimental Section

### 2.1. Study Design

The study was conducted at the Dietetic Outpatient Clinic of the Department of Dietetics, Warsaw University of Life Sciences (WULS-SGGW). The study was carried out according to the guidelines that were laid down in the Declaration of Helsinki and all of the procedures involving human subjects were approved by the Bioethical Commission of the Central Clinical Hospital of the Ministry of Interior in Warsaw (No 35/ 2009) and the Bioethical Commission of the National Food and Nutrition Institute (No 1604/ 2009). Written informed consent was provided by all participants.

### 2.2. Study Participants

The study was carried out on Caucasian male and female individuals with ulcerative colitis in remission. The participants were recruited and monitored at the following Warsaw Gastroenterology Outpatient Clinics: Gastroenterology Outpatient Clinic in Maria Skłodowska-Curie Memorial Cancer Center and Institute of Oncology, Gastroenterology Outpatient Clinic in Central Clinical Hospital of the Ministry of Interior in Warsaw, and Gastroenterology Outpatient Clinic in Public Central Teaching Hospital in Warsaw.

A total of 56 Caucasian individuals with ulcerative colitis in remission (19 males and 37 females) were recruited for the study (Table 1). Inclusion criteria were the same as in the previously published studies [21,22]: nonhospitalized patients with endoscopically diagnosed ulcerative colitis and with confirmed remission (endoscopic remission: image without any changes or disappearance of vascular network, erythema, inflammatory polyps allowed; clinical remission: assessed based on the Mayo Scoring System and Rachmilewitz index for the assessment of the ulcerative colitis activity), aged 18–80 years, in clinical remission for a duration of at least six weeks, and on drugs at a constant dose for at least six weeks. For the Mayo Scoring System, the cut-off of two points in a 12-points scale and for the Rachmilewitz index the cut-off of four points in a 31-points scale were chosen to recruit the remission individuals, as it is commonly indicated [23]. Pregnant women, patients with diagnosed cancer, and those who had underwent gastrointestinal resections were excluded. An excessive weight was observed in a number of included participants, as it is typical for individuals with ulcerative colitis in the remission phase [24].

The basic characteristics of the disease observed in the study participants with ulcerative colitis in the remission phase are presented in Table 2. The frequency of concurrent diseases was compared between male and female individuals and it did not differ for diseases of the blood and blood-forming organs (D50–D89), disorders of thyroid gland (E00–E07), diabetes mellitus and other disorders of glucose regulation and pancreatic internal secretion (E10–E16), disorders of lipoprotein metabolism and other lipidaemias (E78), mental and behavioural disorders (F00–F99), diseases of the nervous system (G00–G99), hypertensive diseases (I10–I15), diseases of the respiratory system (J00–J99), diseases of the digestive system other than noninfective enteritis and colitis, as well as other diseases of the intestines (K00–K46; K65–K93), diseases of the skin and subcutaneous tissue (L00–L99), diseases of the musculoskeletal system and connective tissue (M00–M99), inflammatory diseases of female pelvic organs, as well as noninflammatory disorders of female genital tract and other disorders of the genitourinary system (N70–N99, based on International Statistical Classification of Diseases and Related Health Problems (ICD-10) [25]).

The gastrointestinal symptoms of the patients were assessed based on the self-reported data. All of the patients were asked about daily frequency of bowel movements, and the presence of painful tenesmus, flatulence, and constipation during remission of ulcerative colitis. They were asked to compare the frequency and intensity of symptoms before the diagnosis of ulcerative colitis (painful tenesmus, flatulence, and constipation) with their current conditions. Constipation was defined as less than one bowel movement per three days, while the presence of painful tenesmus and flatulence was subjectively assessed by patients. 

The interview protocol for the subjective assessment was identical for all of the patients. The time frame were specified as three independent periods: before the first onset of ulcerative colitis, during the exacerbation of ulcerative colitis and during the remission of ulcerative colitis. Firstly, they were asked about their well-being and specific symptoms before the first onset of the disease and they were asked to characterize all of the symptoms with the leading questions, if needed. The leading questions were as follows: was the specific symptom observed, what was the frequency of observing the symptom, what was the intensity of the symptom assessed on a scale from 0 to 10, were there any additional factors that in a noticeable way influenced the symptoms (including the dietary factors). Afterwards, patients were asked in an identical way about their well-being and specific symptoms during the exacerbation of ulcerative colitis and after, in an identical way about their well-being and specific symptoms during the remission of ulcerative colitis. Afterwards, the patient was asked to summarize the answers for each symptom separately, and finally, he was asked to indicate if his well-being and specific symptoms changed while comparing the situation before the first onset of the disease and the situation during remission (with the leading question suggesting that his well-being may be now better or worse). Lack of painful tenesmus, flatulence, and constipation during remission or the frequency and intensity of the symptoms not higher than that observed before the diagnosis of ulcerative colitis were interpreted as an absence of the mentioned symptoms. The obtained information were precisely based on the subjective observation and the assessment of indicated symptoms by patients, but it must be indicated that some uncertainty also results from the remembrance of patients (in health research so-called recall bias) [26].

The potential interfering influence of medications was also assessed. The sub-groups stratified by the medications applied (in the groups of 5-aminosalicylic acid medications, corticosteroid medications, and immunosuppressive medications) were compared and no significant differences were observed, so the medications that were applied due to ulcerative colitis were stated to not interfere with the observed associations.

### 2.3. Analysis of Diet

The three-day dietary records of patients with ulcerative colitis in remission were collected and analyzed. Due to the fact that the symptoms during the remission of ulcerative colitis were analyzed, the diet was assessed for the same time frame. Assessment of the diets was based on the self-reported data from dietary records and it was conducted in three typical nonconsecutive random days (two weekdays and one weekend day). The dietary record was conducted based on the widely accepted and applied rules [27]. To obtain reliable estimates of food intake, participants were instructed about the principles of maintaining proper dietary record, as well as about the necessity of accurate and scrupulous recording of all consumed food products and beverages, while they were allowed to report either weight in grams (while using a kitchen scale or consuming packed product with such information presented) or size described using a household measures (while not having a kitchen scale). The serving sizes were verified for each participant individually, using the Polish Atlas of Food Products and Dishes Portion Sizes [28]. For each product or dish that was reported in three-day dietary record of patient, he was shown the relevant photographs (three serving sizes: small, medium, and large) and was asked about more detailed description of his serving size in comparison with the photographs. Additionally, the patient was asked about the specific ingredients of dishes, if they were not declared in details. The whole procedure was conducted by a dietitian, as commonly conducted in the nutritional research [29].

The intake of retinoids (total vitamin A (µg retinol activity equivalent), retinol (µg), β-carotene (µg), α-carotene (µg), β-cryptoxanthin (µg), lycopene (µg), and lutein and zeaxanthin (µg)) was assessed using the National Nutrient Database for Standard Reference of the United States Department of Agriculture [30] due to the lack of Polish database. The average intake levels of the analyzed nutrients (mean values from three recorded days) were the basis for further analysis. 

The fiber intake (g) was assessed as an additional variable that may influence the other factors [31], because even individuals in the remission phase may reduce their fiber intake due to the fear of disease complications [32]. This variable was assessed using the Polish dietician software Dieta version 5.0 (National Food and Nutrition Institute, Warsaw, Poland) and the Polish database of the nutritional value of the products [33].

No specific recommendations have been formulated for vitamin A intake for individuals with inflammatory bowel diseases. The content of total vitamin A in the diets of the affected individuals was typical in comparison with the Estimated Average Requirement level that is recommended for healthy men and women (630 µg retinol activity equivalent for males and 500 µg retinol activity equivalent for females) [34]. In the study of Vagianos et al. [13], it was observed that vitamin A intake in 26% of the analyzed inflammatory bowel diseases individuals was lower than recommended, which was comparable with the share of 27% of patients in the own study. For single retinoids (retinol, β-carotene, α-carotene, β-cryptoxanthin, lycopene, as well as lutein and zeaxanthin), no intake recommendations are formulated for either healthy individuals or individuals with ulcerative colitis.

### 2.4. Statistical Analysis

The obtained data are presented as median with minimum and maximum values, due to the nonparametric distribution. The distribution of the analyzed factors was verified using the Shapiro–Wilk test. The differences between groups were analyzed using the *t*-Student test (applied for parametric distribution) and Mann–Whitney *U* test (applied for nonparametric distribution). The analysis of categorized variables was conducted using the chi^2^ test. The analysis of correlation was conducted using Spearman’s rank correlation (applied for nonparametric distribution). The level of significance *p* ≤ 0.05 was accepted. The statistical analysis was carried out using Statistica software version 8.0 (StatSoft Inc, Tulsa, OK, USA). 

## 3. Results

During the assessment of the daily number of bowel movements, the participants reported that they had high frequency of defecation (ranging from one to nine bowel movements, with a median of three; nonparametric distribution). The vast majority of them reported that they had two to four bowel movements per day (two bowel movements: 45%, three bowel movements: 27%, and four bowel movements: 18%). The analysis of correlation between the levels of retinoid intake and the daily number of bowel movements in the patients with ulcerative colitis revealed no statistically significant association for any of the analyzed retinoids.

Subsequently, the retinoid intake in the participants was analyzed in reference to declared painful tenesmus (18% of assessed individuals), flatulence (16% of assessed individuals), and constipation (13% of assessed individuals).

**Figure 1:**
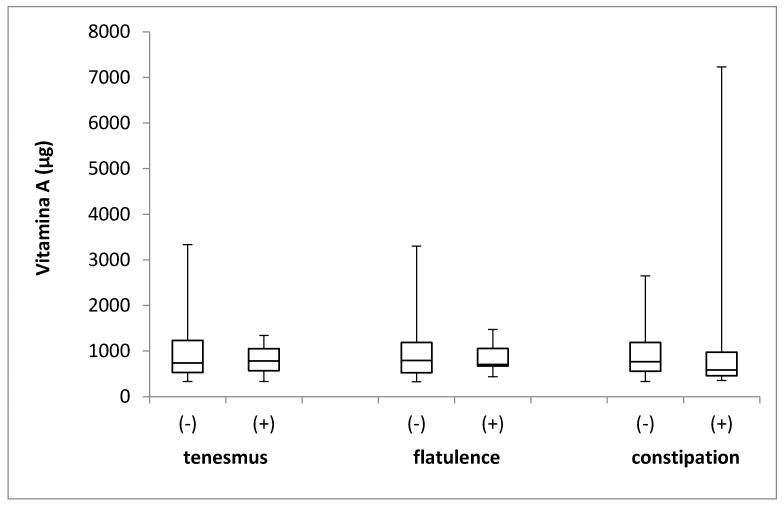
The box plots of vitamin A intake in groups of individuals stratified by the analyzed symptoms of tenesmus, flatulence and constipation. (-) symptom not declared; (+) symptom declared.

The vitamin A intake in the groups of individuals that were stratified by the incidence of tenesmus, flatulence, and constipation is presented in Figure 1. No differences in the levels of vitamin A intake were identified between the groups, with respect to the incidence of tenesmus (*p* = 0.740; Mann–Whitney *U* test), flatulence (*p* = 0.947; Mann–Whitney *U* test), and constipation (*p* = 0.742; Mann–Whitney *U* test).

**Figure 2:**
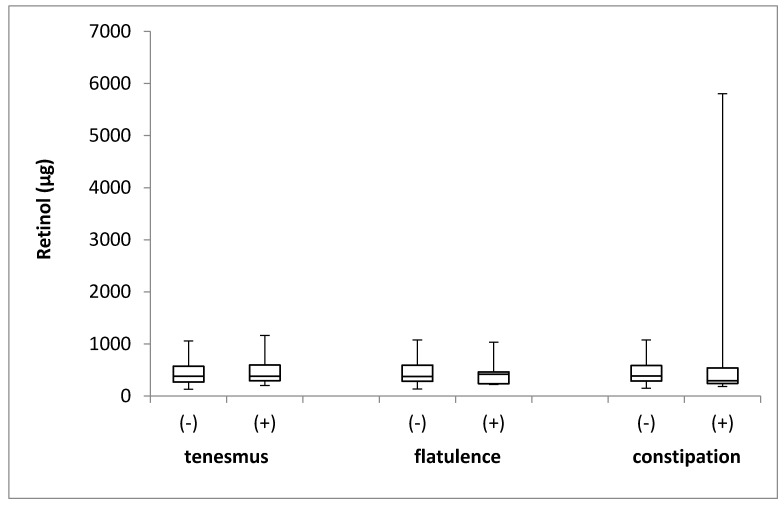
The box plots of retinol intake in groups of individuals stratified by the analyzed symptoms of tenesmus, flatulence and constipation. (-) symptom not declared; (+) symptom declared.

The retinol intake in the groups of individuals stratified by the incidence of tenesmus, flatulence, and constipation is presented in Figure 2. No differences in the levels of retinol intake were identified between the groups, with respect to the incidence of tenesmus (*p* = 0.740; Mann–Whitney *U* test), flatulence (*p* = 0.964; Mann–Whitney *U* test), and constipation (*p* = 0.674; Mann–Whitney *U* test).

**Figure 3:**
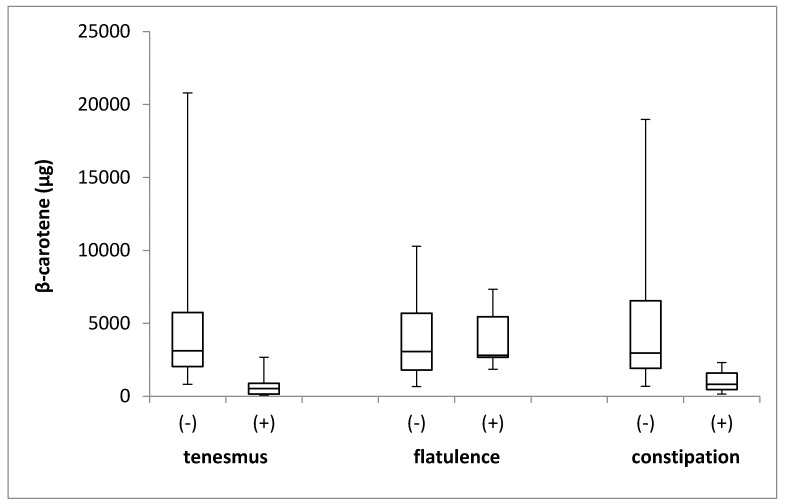
The box plots of β-carotene intake in groups of individuals stratified by the analyzed symptoms of tenesmus, flatulence and constipation. (-) symptom not declared; (+) symptom declared.

The β-carotene intake in the groups of individuals stratified by the incidence of tenesmus, flatulence, and constipation is presented in Figure 3. No differences in the levels of β-carotene intake were identified between the groups, with respect to the incidence of tenesmus (*p* = 0.181; Mann–Whitney *U* test), flatulence (*p* = 0.842; Mann–Whitney *U* test), and constipation (*p* = 0.638; Mann–Whitney *U* test).

**Figure 4:**
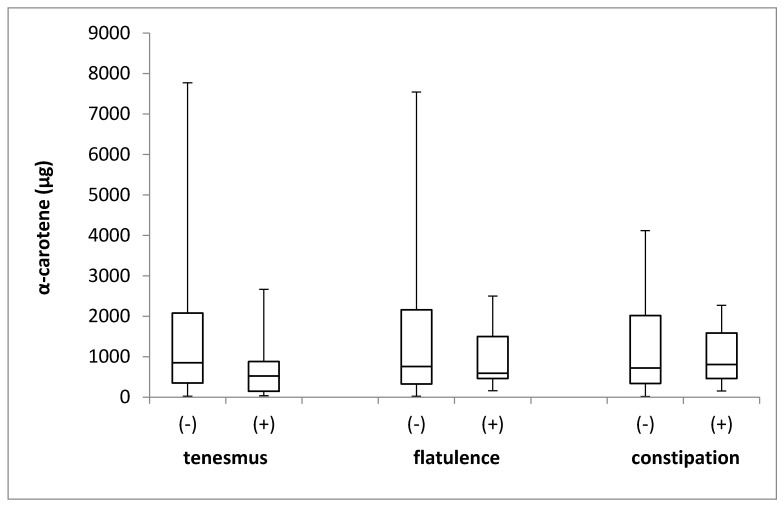
The box plots of α-carotene intake in groups of individuals stratified by the analyzed symptoms of tenesmus, flatulence and constipation. (-) symptom not declared; (+) symptom declared.

The α-carotene intake in the groups of individuals stratified by the incidence of tenesmus, flatulence, and constipation is presented in Figure 4. No differences in the levels of α-carotene intake were identified between the groups, with respect to the incidence of tenesmus (*p* = 0.244; Mann–Whitney *U* test), flatulence (*p* = 0.858; Mann–Whitney *U* test), and constipation (*p* = 0.980; Mann–Whitney *U* test).

**Figure 5:**
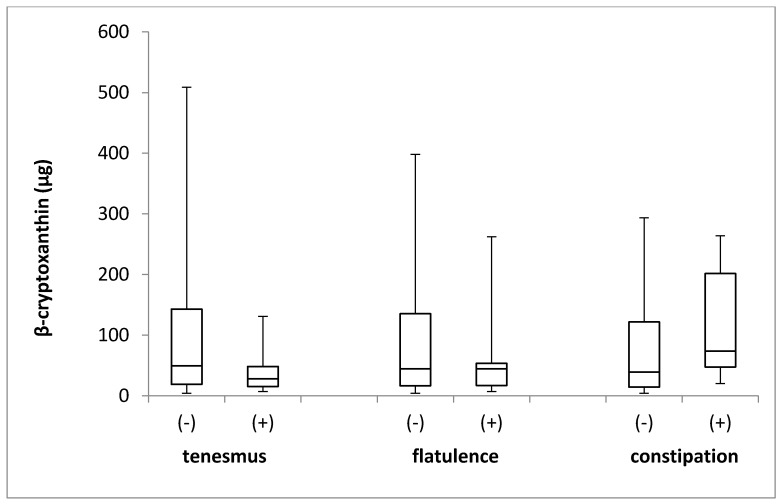
The box plots of β-cryptoxanthin intake in groups of individuals stratified by the analyzed symptoms of tenesmus, flatulence and constipation. (-) symptom not declared; (+) symptom declared.

The β-cryptoxanthin intake in the groups of individuals stratified by the incidence of tenesmus, flatulence, and constipation is presented in Figure 5. No differences in the levels of β-cryptoxanthin intake were identified between the groups, with respect to the incidence of tenesmus (*p* = 0.227; Mann–Whitney *U* test), flatulence (*p* = 0.806; Mann–Whitney *U* test), and constipation (*p* = 0.181; Mann–Whitney *U* test).

**Figure 6:**
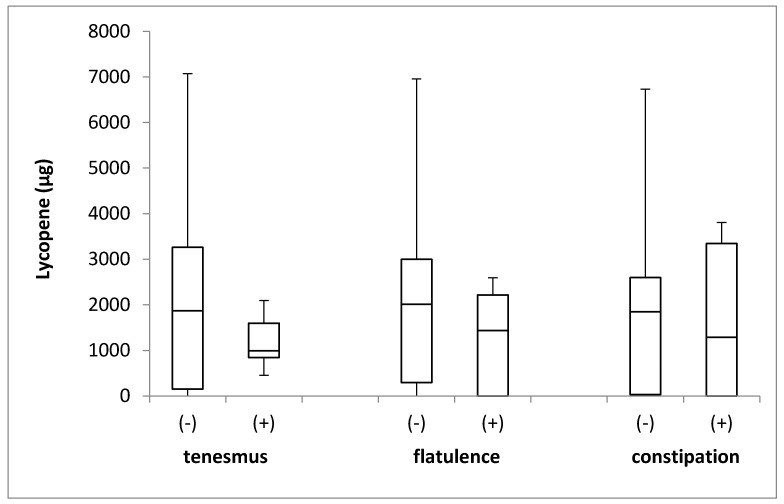
The box plots of lycopene intake in groups of individuals stratified by the analyzed symptoms of tenesmus, flatulence and constipation. (-) symptom not declared; (+) symptom declared.

The lycopene intake in the groups of individuals stratified by the incidence of tenesmus, flatulence, and constipation is presented in Figure 6. No differences in the levels of lycopene intake were identified between the groups, with respect to the incidence of tenesmus (*p* = 0.290; Mann–Whitney *U* test), flatulence (*p* = 0.216; Mann–Whitney *U* test), and constipation (*p* = 0.785; Mann–Whitney *U* test).

**Figure 7:**
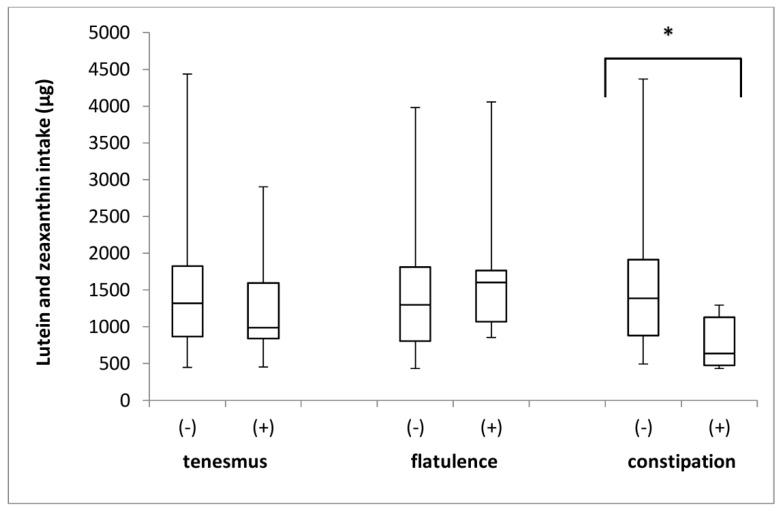
The box plots of lutein and zeaxanthin intake in groups of individuals stratified by the analyzed symptoms of tenesmus, flatulence and constipation. (-) symptom not declared; (+) symptom declared.

The lutein and zeaxanthin intake in the groups of individuals stratified by the incidence of tenesmus, flatulence, and constipation is presented in Figure 7. No differences in the intake of lutein and zeaxanthin were identified between the groups, with respect to the incidence of tenesmus (*p* = 0.760; Mann–Whitney *U* test) and flatulence (*p* = 0.294; Mann–Whitney *U* test). However, significant differences in intake were found between the groups with respect to the incidence of constipation (*p* = 0.013; Mann–Whitney *U* test).

**Figure 8:**
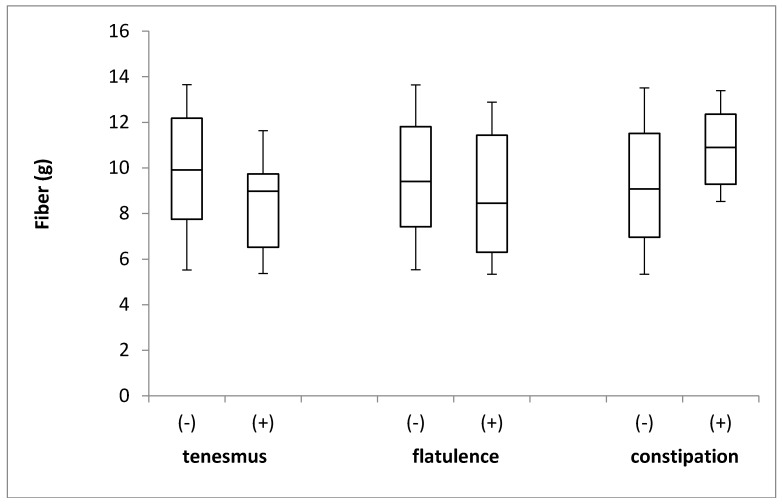
The box plots of fiber intake in groups of individuals stratified by the analyzed symptoms of tenesmus, flatulence and constipation. (-) symptom not declared; (+) symptom declared.

The fiber intake in the groups of individuals stratified by the incidence of tenesmus, flatulence, and constipation is presented in Figure 8. No differences in the levels of fiber intake were identified between the groups, with respect to the incidence of tenesmus (*p* = 0.231; Mann–Whitney *U* test), flatulence (*p* = 0.514; Mann–Whitney *U* test), and constipation (*p* = 0.448; Mann–Whitney *U* test).

The results of the analysis of correlation between fiber intake and retinoids intake are presented in Table 3. No significant association was found for any of the assessed retinoids. 

## 4. Discussion

### 4.1. Constipation in Individuals with Inflammatory Bowel Disease

Based on the analysis of the self-reported data on gastrointestinal symptoms of individuals with ulcerative colitis, it was found that a significant association existed only between constipation and retinoid intake.

Constipation is not a typical symptom of ulcerative colitis [35]. While diarrhea is common, chronic constipation without diarrhea may sometimes occur as an unusual presentation of the disease [36]. However, in some individuals with ulcerative colitis, mixed symptoms are also observed, as patients simultaneously declare an increased frequency of defecation and constipation [37].

In the research of Gearry et al. [38], constipation was identified as a symptom of the disease that is not modified by reducing the intake of short-chain carbohydrates, both in individuals with ulcerative colitis and individuals with Crohn’s disease. The modified diet (FODMAP-restricted diet—Fermentable Oligo-Di-Monosaccharides and Polyols-restricted diet) that was mentioned in a systematic review was observed to be effective only for short-term treatment of selected patients with irritable bowel syndrome and constipation [39]. The possible application of other diet modifications for the reduction of constipation has not been tested in individuals with inflammatory bowel diseases thus far. Crohn’s and Colitis UK recommends increasing the intake of fluids and fiber for these individuals, but it has been indicated that a high fiber intake will not be beneficial for all of the patients with inflammatory bowel diseases [40]. However, high fiber intake is considered as especially important, as it may increase the production of short-chain fatty acids, which are believed to have therapeutic potential [41].

On the other hand, dietary modifications for treating constipation in individuals with inflammatory bowel diseases may not always be effective. This is because fiber intake does not diminish painful defecation and it may not reduce constipation in these individuals. Constipation that is observed in the case of individuals with inflammatory bowel diseases may result from their subconscious attempts to minimize pain during bowel movements, which may be associated with an altered pattern of abdominal wall and the activation of pelvic and sphincteric muscles [42]. Simultaneously, it has been observed that patients with inflammatory bowel diseases are characterized by lower intake of fluids and fiber than healthy individuals [43], so even recommended increase in fluids and fiber intake may not effectively modify the diet of individuals with ulcerative colitis. 

It should also be noted that the low intake of fluids and fiber might contribute to the development of inflammatory bowel diseases, as it leads to a decrease in the volume of colonic content, as well as increase in colonic concentration of toxic compounds and mucosal exposition on them [44]. However, it cannot be confirmed that only these mentioned factors are associated with the incidence of inflammatory bowel diseases, as the same observation was also noted for retinol intake [43]. 

### 4.2. Role of Vitamin A in Individuals with Inflammatory Bowel Disease

Andersen et al. [45] hypothesized that vitamin A may exert a significant influence on the intestinal inflammation and protect the intestine from pathogens via initiating the immune response. It was indicated that metabolites of vitamin A trigger dendritic cells to activate regulatory T cells, while the lack of such metabolites stimulates dendritic cells to induce inflammation-promoting Th17 cells. Simultaneously, it was indicated that retinoic acid takes part in the translocation of antigen-presenting B and T cells to the gut [45]. This corresponds with the general observation that serum carotenoids are inversely associated with inflammatory markers [46].

Gastrointestinal symptoms may be especially associated with vitamin A intake, which is confirmed by the present study. In addition, in the research of Nagórna-Stasiak and Wawrzeńska [47], it was observed that vitamin A and vitamin D, but not vitamin E, influenced the intestinal motility in the animal model. This might be due to the fact that retinoic acid increases neuronal differentiation [48]. It must be emphasized that the constipations incidence observed in the presented own study is only one of elements of intestinal motility. Moreover, constipations incidence may be related to motility, but also may result from a number of other reasons that are not related to motility per se. Among such factors, there are some diet-related ones, such as fiber intake, which in the presented own study was excluded as a potential reason, due to a lack of significant association with the assessed symptoms.

In another study, the enteric neurons developing in the retinoid-deficient conditions were found to have neurites of significantly lower length when compared to those developing in the presence of high retinoic acid concentration [49]. This indicated that vitamin A forms may play an important role not only in constipation, as observed in the present study, but also in other diseases, including Hirschsprung’s disease [49], due to their association with enteric nervous system [50].

### 4.3. Role of Lutein and Zeaxanthin in Individuals with Inflammatory Bowel Disease

The influence of intake of retinoids, which are a chemical form of vitamin A, may vary. Lutein and zeaxanthin are the only forms of vitamin A that affect constipation frequency, as was observed in the present study. This may be attributed to the fact that lutein is a powerful antioxidant that can decrease intestinal oxidation [51,52] and prevent intestinal damage [53,54]. In the animal model of inflammation, lutein was found to be the only retinoid to selectively inhibit the activation of transient receptor potential ankyrin 1 (TRPA1) and the resultant inflammation [55]. Additionally, lutein and zeaxanthin were observed to modulate proinflammatory cytokines (IL-1β, IL-6, and IFN-γ) in the liver, duodenum, jejunum, and ileum, as well as anti-inflammatory cytokine (IL-10) in the liver and jejunum in the animal model [56].

Based on the results of the mentioned research, it may be suggested that the intake of lutein and zeaxanthin is a factor influencing the inflammatory process and the course of the inflammatory bowel diseases, and thus it may also modify several symptoms of the disease, such as the incidence of constipation. In the present study, the intake of lutein and zeaxanthin was not modified by fiber intake, as no significant difference was found between groups declaring constipation and declaring lack of constipation. Moreover, a previous analysis that was conducted in the studied group revealed that lycopene, lutein, and zeaxanthin might also reduce the incidence of fecal blood, mucus, and pus [20].

In the present study, an association was only observed between the intake of lutein and zeaxanthin and constipation. This observation is especially important as decreased levels of lutein and zeaxanthin are identified in general in individuals with inflammatory bowel diseases. In the research of Mózsik et al. [57], the total blood levels of vitamin A and zeaxanthin were found to be lower than expected in the patients with inflammatory bowel diseases. Similarly, in the study of Rumi et al. [58], the blood levels of total vitamin A, lutein, and zeaxanthin were significantly lower in patients with inflammatory bowel diseases when compared to healthy participants.

### 4.4. Possible Recommendations for Individuals with Inflammatory Bowel Disease

Taking into account the numerous studies indicating the role of lutein and zeaxanthin in the course of inflammatory bowel diseases, it may be suggested that these nutrients have a substantial anti-inflammatory effect. Moreover, as very low levels of lutein and zeaxanthin are observed in individuals with inflammatory bowel diseases, the recommendation of a higher intake of these nutrients could be of a great value. Based on the results of the present research, it can be concluded that modification of diet may be beneficial especially in the case of individuals suffering from constipation. 

Practical guidelines regarding dietary habits may be suggested for the individuals with inflammatory bowel diseases to reduce the incidence of constipation and the resultant discomfort. Foods that are rich in lutein, especially vegetables, which are also quite rich in fiber, should be recommended. This is also in agreement with the general recommendations for individuals with inflammatory bowel disease [59]. Appropriate food products should be chosen instead of other fiber sources that are sometimes avoided in diet by individuals with inflammatory bowel diseases. 

As vegetables are the main source of lutein, not only in the Polish diet [60], but also in the diet of other western countries [61], they should be recommended in order to minimize the negative symptoms of disease. If well tolerated, spinach (8950 µg of lutein per serving), pumpkin (2820 µg), green peas (2230 µg), broccoli (1970 µg), young beetroot leaves (1960 µg), celery (1680 µg), and zucchini (1140 µg) should be included in diet to increase lutein levels [62].

The recent review study by Oliveira et al. [63] emphasized the role of retinoids for inflammatory diseases, and particularly, for the inflammatory bowel diseases, as well as it was indicated that more studies are necessary for better understanding of role of retinoids in inflammatory processes. Taking it into account, all the relevant analysis, even if their findings do not specify the association, may enable the future understanding. Especially, the studies of the potential influence of retinoids on the constipation incidence may be valuable, due to the suggested role of retinoids in the prevention of colon cancer [1] and the commonly studied association between constipations and colon cancer [64]. Taking it into account, it may be suggested that the intake of specific retinoids may be especially valuable for inflammatory bowel diseases patients as a complementary treatment, due to a comprehensively beneficial potential effect and further studies are needed to obtain the important public health purpose.

### 4.5. Limitations of the Study 

This study was conducted in a relatively small group of individuals with ulcerative colitis in the remission phase. In spite of the fact that the group was homogenic, taking into account the remission phase, the symptoms of the disease, and concurrent diseases, there is a limited possibility to generalize the results to patients with inflammatory bowel disease. Moreover, in the present study, only limited data were assessed, as retinoids were the main objective of the study and not all nutrients were analyzed.

The patients’ diet was assessed based on their three-day dietary record, and the assessment was conducted during nonconsecutive days. This is the most commonly applied method of assessment of diet and recommended by a number of institutions, including the National Institutes of Health/National Cancer Institute [65]. However, the limitation that is associated with this method must also be indicated, as researchers never know whether the obtained record is really reliable and even longer records do not guarantee it. 

Another limitation of the study was multiple comparisons, as the observed levels of retinoid intake were not independent variables but all resulted from the same diet. The possibility of influence of other nutrients on the analyzed association was also a limitation. However, this issue is always stated in any nutritional analysis, as it is almost impossible to assess only one nutrient in a diet with no possible influence of the others. In order to minimize the possible interference, all of the retinoids were analyzed and fiber content was included as the most important interfering factor. However, the analysis of correlation between fiber content and retinoids showed that there was no possible influence of fiber content.

## 5. Conclusions

- One in every seven individuals with ulcerative colitis in remission report not only increased frequency of bowel movements, but also recurring constipation.
- Higher intake of lutein and zeaxanthin may be a potential dietary factor to decrease the incidence of constipation in these individuals.
- The present study showed that the intake of retinoids other than lutein and zeaxanthin was not associated with the incidence of constipation or other gastrointestinal symptoms in the case of individuals with ulcerative colitis.

## Figures and Tables

**Table 1:** The basic characteristics of the group of individuals with ulcerative colitis in remission phase recruited for the study.

		Total (*n* = 56)	Male Individuals (*n* = 19)	Female Individuals (*n* = 37)	*p*-Value
Age (years)		47.4 ± 13.7 48.0 (19.0–69.0)	45.9 ± 13.9 45.0 (23.0–69.0)	48.2 ± 13.6 49.0 (19.0–68.0)	0.5614 ^a^
Weight (kg)		76.7 ± 15.7 76.5 (48.0–115.0)	84.6 ± 12.9 80.0 (69.0–112.0) *	72.7 ± 15.6 72.0 (48.0–115.0)	0.0051 ^b^
Height (cm)		168.2 ± 8.9 167.0 (153.0–186.0)	177.7 ± 6.4 180.0 (166.0–186.0)	163.3 ± 5.2 165.0 (153.0–175.0)	0.0000 ^a^
Body Mass Index (BMI)	(kg/m^2^)	27.1 ± 5.1 26.7 (17.6–38.1)	26.8 ± 3.9 26.3 (20.2–34.6)	27.3 ± 5.7 27.7 (17.6–38.1)	0.7437 ^a^
	Underweight	3 (5.4%)	0 (0.0%)	3 (8.1%)	0.3026 ^c^
	Normal weight	18 (32.1%)	6 (31.6%)	12 (32.4%)	
	Overweight	19 (33.9%)	9 (47.4%)	10 (27.1%)	
	Obesity	16 (28.6%)	4 (21.0%)	12 (32.4%)	

* nonparametric distribution (verified using the Shapiro–Wilk test; *p* ≤ 0.05); ^a^ compared using *t*-Student test based on mean values (applied due to parametric distributions); ^b^ compared using Mann–Whitney *U* test based on median values (applied due to non-parametric distributions); ^c^ compared using chi^2^ test.

**Table 2:** The basic characteristics of the disease course for a group of individuals with ulcerative colitis in remission phase recruited for the study.

		Total (*n* = 56)	Male Individuals (*n* = 19)	Female Individuals (*n* = 37)	*p*-Value
Treatment duration (years)		7.1 ± 3.3 7.0 (2–17)	6.5 ± 3.0 7.0 (2–13)	7.4 ± 3.4 6.5 (2–17) *	0.5711 ^a^
Severity of exacerbations **	Mild	7 (12.5%)	4 (21.0%)	3 (8.1%)	0.0124 ^b^
	Moderate	46 (82.1%)	12 (63.2%)	34 (91.9%)	
	Severe	3 (5.4%)	3 (15.8%)	0 (0.0%)	
Hospitalizations frequency (number per year)		0.28 ± 0.20 0.29 (0–0.73)	0.29 ± 0.22 0.29 (0–0.63)	0.28 ± 0.19 0.29 (0–0.73) *	0.7838 ^a^
Concurrent diseases ***	(number of diagnosed ones)	2.9 ± 2.0 3.0 (0.0–8.0)	2.5 ± 2.1 2.0 (0.0–8.0)	3.1 ± 1.9 3.0 (0.0–8.0)	0.3547 ^c^
	D50–D64	16 (28.6%)	7 (36.9%)	9 (24.3%)	0.5032 ^b^
	E00–E07	13 (23.2%)	1 (5.3%)	12 (32.4%)	0.0517 ^b^
	E10–E16	3 (5.4%)	1 (5.3%)	2 (5.4%)	1.0000 ^b^
	E78	3 (5.4%)	1 (5.3%)	2 (5.4%)	1.0000 ^b^
	F00–F99	8 (14.3%)	1 (5.3%)	7 (18.9%)	0.3274 ^b^
	G00–G99	3 (5.4%)	0 (0.0%)	3 (8.1)	0.5163 ^b^
	I10–I15	15 (26.8%)	3 (15.8%)	12 (32.4%)	0.3111 ^b^
	J00–J99	4 (7.1%)	2 (10.5%)	2 (5.4%)	0.8765 ^b^
	K00–K46; K65–K93	28 (50.0%)	10 (52.6%)	18 (48.6%)	1.0000 ^b^
	L00–L99	8 (14.3%)	3 (15%)	5 (13.5%)	1.0000 ^b^
	M00–M99	20 (35.7%)	5 (26.3%)	15 (40.5%)	0.4489 ^b^
	N70–N99	4 (7.1%)	0 (0.0%)	4 (10.8%)	0.3476 ^b^

* nonparametric distribution (verified using the Shapiro–Wilk test; *p* ≤ 0.05); ** assessed by attending gastroenterologist on the basis of the recorded data gathered since the moment of diagnosis; *** based on International Statistical Classification of Diseases and Related Health Problems (ICD-10) [25], for: diseases of the blood and blood-forming organs (D50–D89), disorders of thyroid gland (E00–E07), diabetes mellitus and other disorders of glucose regulation and pancreatic internal secretion (E10–E16), disorders of lipoprotein metabolism and other lipidaemias (E78), mental and behavioural disorders (F00–F99), diseases of the nervous system (G00–G99), hypertensive diseases (I10–I15), diseases of the respiratory system (J00–J99), diseases of the digestive system other than noninfective enteritis and colitis as well as other diseases of the intestines (K00–K46; K65–K93), diseases of the skin and subcutaneous tissue (L00–L99), diseases of the musculoskeletal system and connective tissue (M00–M99), inflammatory diseases of female pelvic organs, as well as noninflammatory disorders of female genital tract and other disorders of the genitourinary system (N70–N99); ^a^ compared using Mann–Whitney *U* test based on median values (applied due to non-parametric distributions); ^b^ compared using chi^2^ test; ^c^ compared using *t*-Student test based on mean values (applied due to parametric distributions).

**Table 3:** Analysis of correlation (Spearman’s rank correlation) between fiber intake and retinoid intakes.

	*p*-Value	R
Vitamin A	0.782	0.0377
Retinol	0.322	−0.1347
β-carotene	0.544	0.0828
α-carotene	0.186	0.1792
β-cryptoxanthin	0.841	0.0274
Lycopene	0.776	0.0388
Lutein and zeaxanthin	0.859	0.0242
